# Self-Adhesive, Human Bandage Contact Lens Versus Conjunctival Transposition Flap for Surgical Repair of Feline Corneal Sequestrum

**DOI:** 10.3390/vetsci12090839

**Published:** 2025-08-30

**Authors:** Khaled M. Ali, Ayman A. Mostafa

**Affiliations:** 1 Department of Small Animal Surgery and Ophthalmology, Faculty of Veterinary Medicine, Cairo University, Giza 12613, Egypt; 2 Department of Veterinary Clinical Sciences, College of Veterinary Medicine, Western University of Health Sciences, Pomona, CA 91766, USA

**Keywords:** self-adhesive, human bandage, contact lens, feline, corneal sequestrum, conjunctival flap, feline ophthalmology

## Abstract

Corneal sequestrum, also known as corneal mummification or necrosis, is a common complication of corneal ulceration in cats, with brachycephalic and pure-bred cats being the most affected. This prospective clinical trial aimed to evaluate the efficacy of self-adhesive, human bandage contact lenses (SHBCLs) versus the commonly used conjunctival transposition flap (CTF) in the management of feline corneal sequestrum. Twenty-five client-owned cats of different breeds with unilateral corneal sequestrum were investigated. All cats underwent a complete ophthalmic examination. Characterization of corneal sequestrum was carried out. The corneal lesion was removed surgically via keratectomy, and the cats were then categorized into three treatment groups: G-SHBCL (10 cats), G-CTF (10 cats), and the control group (G-CO, 5 cats). The healing progress of the corneal defect and associated clinical findings were recorded in all studied groups. Eight out of ten cats (80%) treated with SBCLs showed complete healing of the corneal defect with light corneal scarring and absence of all signs of ocular pain or discomfort. In comparison with G-CTF and G-CO, no granulation tissue was observed in all cats treated with SHBCLs. Moreover, no residues of corneal sequestrum or adhesion were identified, and the degree of corneal clarity was higher in the SHBCL treatment group. In conclusion, application of SHBCLs on a corneal defect, after removal of the associated sequestrum, enhances healing of the cornea and markedly improves the corneal clarity and transparency within a short period.

## Simple Summary

Corneal sequestrum frequently occurs as a complication of corneal ulceration in cats. The aim of this study was to assess the effectiveness of self-adhesive human bandage contact lenses (SHBCLs) in comparison to the traditional conjunctival transposition flap (CTF) for treating feline corneal sequestrum. A total of 25 client-owned cats from various breeds that exhibited unilateral corneal sequestrum were included. Each cat underwent a thorough ophthalmic examination, and the corneal sequestrum was characterized. The corneal lesions were surgically excised through keratectomy, after which the cats were divided into three treatment groups: G-SHBCL, G-CTF, and the control group (G-CO). The healing process of the corneal defect and related clinical observations were documented for all groups under study. In the G-SHBCL group, 80% achieved complete healing of the corneal defect, demonstrating minor corneal scarring and no signs of ocular pain or discomfort. When compared to G-CTF and G-CO, no granulation tissue was found in any of the cats treated with SHBCL. Additionally, there were no remnants of corneal sequestrum or adhesions detected, and the clarity of the cornea was significantly better in the SHBCL treatment group. Applying SHBCL on a corneal defect, following the removal of the associated sequestrum, promotes better healing of the cornea while greatly enhancing corneal clarity and transparency in a short timeframe.

## 1. Introduction

Corneal sequestrum, also known as corneal mummification or necrosis, is a common complication of corneal ulceration in cats [1,2,3], with brachycephalic and pure-bred cats being the most affected [4]. The disease is characterized by the formation of brown lesions in the corneal epithelium and anterior stroma. The real cause of the disease is still unknown [3]; however, several contributing factors have been incriminated in the condition [5], such as chronic corneal irritation, frequent usage of topical corticosteroids [1], and feline herpesvirus type-1 (FHV-1) infection [6]. *Chlamydophila felis* and *Toxoplasma gondii* have also been considered as triggering agents evoking corneal ulcers and sequestrum [6,7]. A previous study excluded the atrophy of the goblet cells and tear film abnormalities from the pathogenesis of corneal sequestrum in cats [8]. The disease is characterized by the formation of a well-defined brown-to-dark lesion in the central or paracentral corneal epithelium and anterior stroma [3]. The size and shape of the lesion and the intensity of the discoloration vary according to the severity and duration of the disease [5]. The origin and nature of these discolorations are still not well-known. A previous study has proven that such discoloration is not due to melanin [6]. Iron and copper have been detected in corneal sequestra and may contribute to tissue degeneration through oxidative stress or pigment accumulation, potentially playing a role in the pathogenesis of feline corneal sequestrum [9]. In humans, corneal discoloration has been attributed to the long-term use of topical epinephrine, which is metabolized into adrenochrome and eventually stable melanin pigment, resulting in ‘black cornea’. While epinephrine is not implicated in cats, this comparison highlights how oxidative conversion of compounds may result in pigment accumulation, potentially analogous to what is observed in feline corneal sequestrum [10].

Ultrastructure components of corneal sequestra were described previously as a large area of necrosed, cellular stromal collagen with multiple inflammatory cells and keratocytes [6]. Corneal sequestrum does not impede the vision of the affected eye except when it is large and occupying the central cornea [11].

In veterinary ophthalmology, many surgical techniques have been described to treat corneal sequestrum in cats. Keratectomy is usually used to expedite the extrusion of irritating, superficial sequestra [12,13]. In case of deep lesions, porcine small intestinal submucosal (SIS) graft [14], amniotic membrane transplantation (AMT) [2], and heterologous penetrating keratoplasty are the commonly used techniques to restore the structural integrity of the affected cornea and achieve minimal alteration to the corneal transparency [2]. Therapeutic contact lenses are those that are applied specifically for the treatment of corneal diseases in humans [15]. Soft contact bandage lenses have also been used in small animal practice [16,17,18], with hydrophilic contact lenses being used in dogs and cats for the treatment of superficial and recurrent corneal erosions [16]. They have since been used extensively as an adjunct therapy to provide comfort and protection via reducing pain and maintaining the tear film over the ulcerated corneal region, thereby promoting healing [17,18]. However, to our knowledge, the use of bandage contact lenses as adjunctive therapy for corneal sequestrum in cats has not been reported in the peer-reviewed veterinary literature to date. The use of contact lenses on corneal defects can remove any associated necrotic tissue and tissue residues by adhering them to the posterior lens surface. This is expected to prevent further destruction and melting of the cornea by the tissue-destructing enzymes, thereby allowing proper healing [18]. Therefore, the purpose of the present study was to evaluate the use of soft, self-adhesive, commercially available human contact lenses versus the commonly used conjunctival transposition flap to surgically repair feline corneal sequestrum after performing nonpenetrating keratectomy. We hypothesize that the treatment of feline corneal sequestrum using a self-adhesive human bandage contact lens would induce better corneal healing and clearer transparency and subsequent proper quality of vision.

## 2. Materials and Methods

### 2.1. Animals

**Figure 1:**
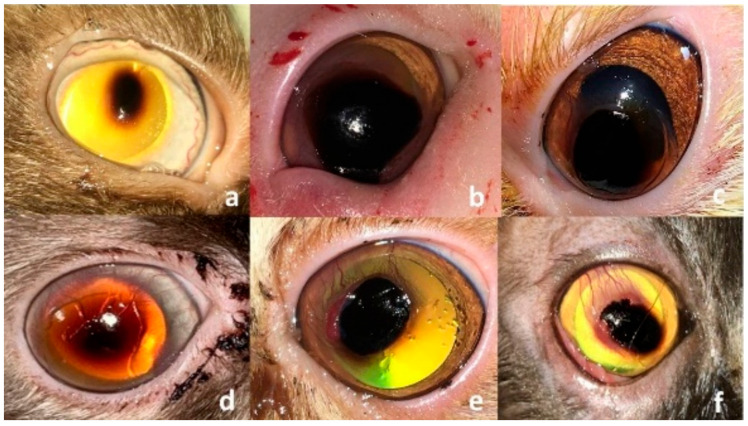
Clinical presentation of corneal sequestrum in different patients. (**a**–**d**), the lesion appears as a small or large, well-defined blackish plaque at the center of the cornea without or with corneal vascularization and ulceration. (**e**,**f**), the lesion may progress to have accompanying granulation tissue and underlying ulceration (**f**).

Twenty-five cats with long-lasting corneal sequestrum (25 affected eyes) were included in the present study. Overall, the breeds included in the present study were Persian (*n* = 12), Himalayan (n = 8) and Domestic shorthaired (DSH, n = 5). The mean age (±SD) at the initial presentation was 37.6 (±16.11) months (range, 12–72 months). There were 13 males (7 intact and 6 castrated) and 12 intact females. The mean (±SD) duration of the clinical signs prior to presentation was 9.2 (±3.6) months (range, 2–14 months). Informed consent was obtained from the owners of all enrolled cats. Data collected from each patient included breed, gender, age, the affected eye, and duration of the clinical signs. Before inclusion, all cats underwent a complete ophthalmic examination including the Schirmer tear test (Ophtechnics Unlimited, Gurugram, India), slit lamp examination (SL 14 handheld slit lamp, Kowa, Tokyo, Japan), fluorescein staining (Bio-Glo^®^ Fluorescein sodium Strips 1 mg; HUB pharmaceuticals, LLC., Scottsdale, AZ, USA) Schiotz tonometry (Riester, Jungingen, Germany) and indirect ophthalmoscopy (Riester, Jungingen, Germany). All ophthalmic examinations were conducted in a quiet indoor clinical setting at a controlled ambient temperature of 22–24 °C. Lighting conditions included dim ambient room lighting, with focused halogen illumination provided by a slit lamp biomicroscope during detailed corneal evaluations. Cases were eligible for inclusion if they received a complete ophthalmic examination and were diagnosed with long-lasting, firmly adhered corneal sequestrum. Patients with corneal sequestrum along with tear film abnormalities or eyelid diseases were excluded. Cases with suspected bacterial keratitis, systemic disease, and/or FHV-1 infection were also excluded. All patients were examined and treated surgically by a qualified ophthalmologist (K.M.A.) working at the College of veterinary medicine, Cairo University between March and December 2022. Cats were diagnosed with corneal sequestrum if the lesion sequestrum appeared as dark brown to black plaque with different shapes and sizes located on the central or paracentral region of the affected cornea [3,5] (Figure 1). The size of the corneal sequestrum was measured using a digital caliper and the morphological characteristics of the sequestrum, in terms of its site, size, depth, and vascularization, were recorded. All study subjects were categorized into three groups: self-adhesive, human bandage contact lens group (G-SHBCL, 10 cats), conjunctival transposition flap group (G-CTF, 10 cats) and control group (G-CO, 5 cats).

### 2.2. Contact Lens

**Figure 2:**
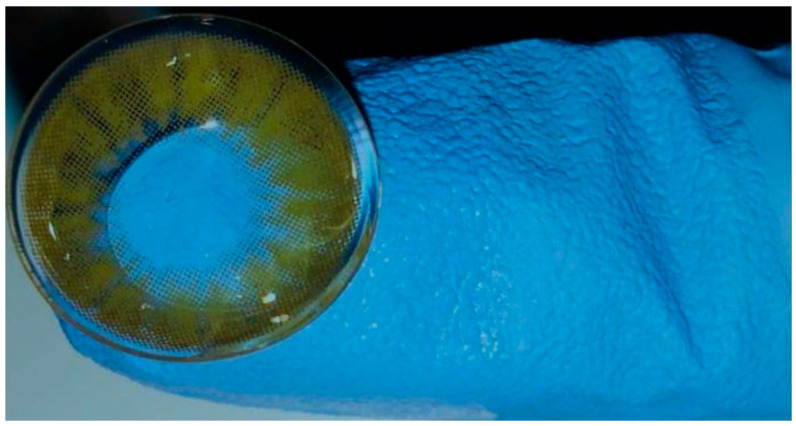
Photograph of the therapeutic, self-adhesive human soft contact bandage lens used in the study.

A commercially available self-adhesive human contact lens (Bella^®^, Inter, JO Co., Ltd., Seoul, Republic of Korea) was used in the present study (Figure 2). The lens material is Polymacon with 38% water content. The inner surface of the lens contains a nanostructure that allows for better dispersion of eye drops and self-purification [15]. The diameter of the utilized lens is 14.5 mm with a lens base curve radius of 8.6 mm and a central thickness of 0.07 mm. The human lens was selected in the study reported here because veterinary lenses designed for cats are not available in Egypt and are much more expensive compared to human lenses.

### 2.3. Surgical Procedure

**Figure 3:**
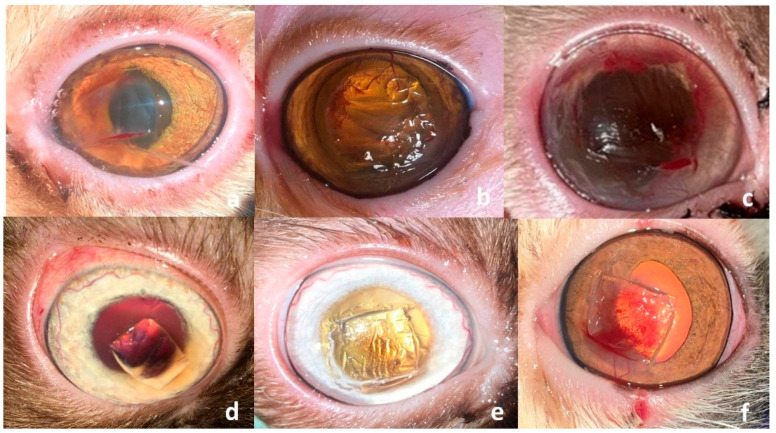
Clinical presentation after successful removal of the corneal sequestrum from different cat breeds enrolled in this study (**a**–**f**). Note the diffuse discoloration that could not be removed surgically in two Himalayan cat (**d**,**e**), and the bleeding after removal of a well-vascularized sequestrum in a Persian cat (**f**).

All cats were premedicated with atropine sulfate (0.04 mg/kg) and xylazine hydrochloride 2% (Xyla ject^®^; ADWIA, New Cairo, Egypt) in a dose of 1 mg/kg *b.w.*, and anesthetized with ketamine hydrochloride 5% (Keiran; EIMC Pharmaceuticals Co., Cairo, Egypt) in a dose of 20 mg/kg. Desensitization of the cornea was achieved before removal of the sequestrum via instillation of Benoxate hydrochloride 0.4% (Benox^®^; EIPICO, 10th of Ramadan, Egypt), with a waiting time of approximately 60 s to ensure adequate corneal anesthesia. All surgical procedures were performed under a binocular surgical microscope (12.5×; 66 VISION TECH Co., Ltd., Shenzhen, China). The eye was prepared for aseptic surgery and draped routinely. A 64 Beaver blade was used to sharply remove the necrotic lesion from the cornea. The dissection was continued through the stromal thickness to a depth that allowed for maximal removal of the sequestrum while preserving healthy adjacent tissue (Figure 3), as previously described [5].

**Figure 4:**
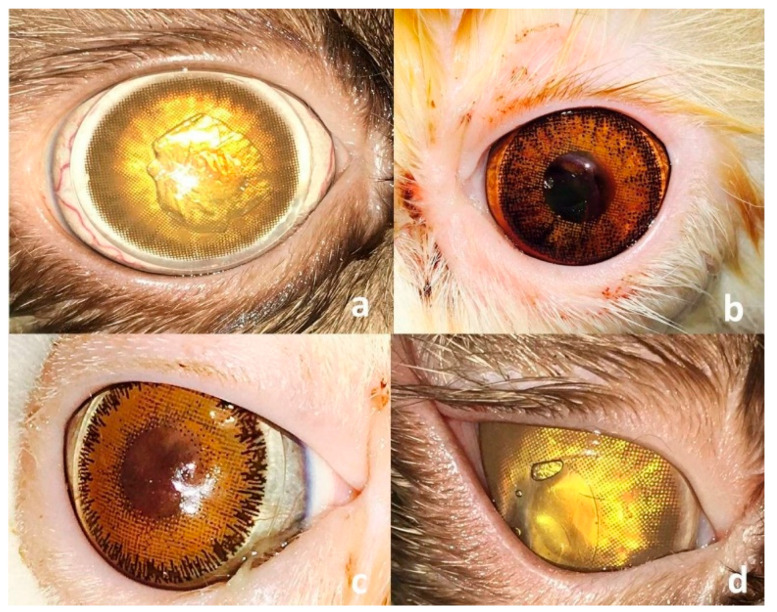
Clinical presentation of four cats immediately after placement of self-adhesive human bandage contact lenses (SHBCL) following keratectomy. The lenses fit well and adhere to the corneas and cover the corresponding corneal defects in Himalayan (**a**), DSH (**b**), and Persian (**c**) cats. Note the temporary tarsorrhaphy stitch that was applied to reduce the palpebral fissure in a Himalayan cat (**d**).

For the cases enrolled under G-SHBCL, a 14.5 mm self-adhesive contact lens was placed onto the corneal defect (Figure 4). The owners were advised to remove and clean the lens using a cleansing solution containing sodium chloride, tetronic surfactant, and sodium phosphate (Cliwell^®^; BESCON Co., Ltd., Pyeongtaek-si, Republic of Korea) every other day. Patients with wide-open eyes (Himalayan, n = 2) had a temporary lateral tarsorrhaphy stitch placed into the affected eye to narrow the corresponding palpebral fissure and provide more fixation to the contact lens (Figure 4d). In the G-CTF, a triangular to rectangular conjunctival flap was created in the bulbar conjunctiva. Steven’s tenotomy scissors were utilized with the base of the flap being attached to the limbus. The length of the flap was adjusted to cover the corneal defect without tension.

**Figure 5:**
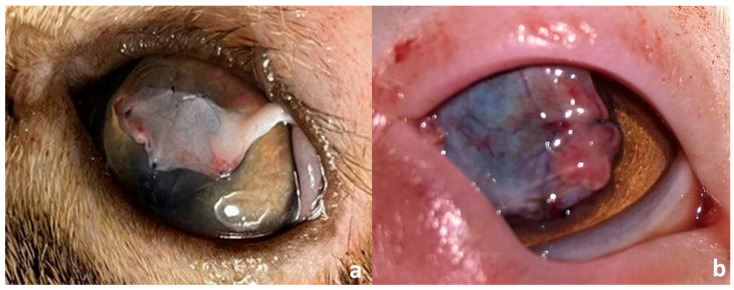
(**a,b**) Clinical presentation of a cat immediately after conjunctival transposition flap (CTF) technique following keratectomy. The conjunctival flap is fixed to the corneal defect margin by interrupted sutures to cover the corneal defect.

The flap was then sutured to the borders of the corneal defect using 8-0 monofilament polypropylene and a spatulated, double, 7 mm ½ circle needle (prolene^®^; Ethicon, Raritan, NJ, USA) in a simple interrupted pattern (Figure 5a,b). The five cats in the control group (G-CO) were treated only by lamellar keratectomy. All patients enrolled in the three groups received postoperative tobramycin eyedrops (Tobrin^®^; EIPICO, 10th of Ramadan, Egypt). The tobramycin medication dosage was decreased and discontinued as healing progressed. All cats received meloxicam (Mobic^®^; Boehringer Ingelheim, Rhein, Germany) at a dose rate of 0.05 mg/kg PO q24h during the first three days after surgery. Owners were asked to bring their cats for a recheck on days 7, 14, and 30 postoperatively.

### 2.4. Statistical Analysis

Data were analyzed using SPSS Statistics for Windows, Version 26.0 (IBM Corp., Armonk, NY, USA). Continuous variables were expressed as mean ± standard deviation (SD). Categorical variables, including treatment success rates between the SHBCL and CTF groups, were compared using Fisher’s Exact Test due to the relatively small sample size in each group. A *p*-value < 0.05 was considered statistically significant.

## 3. Results

### 3.1. Clinical Findings

The sequestra appeared as well-defined, circumscribed dark brown to black plaques on the central (17 cats) or paracentral (8 cats) cornea (Figure 1). The mean (±SD) diameter of the presented sequestra was 5.5 (±1.2) mm (range, 4–8 mm). Corneal sequestrum was diagnosed in 14 right eyes and 11 left eyes, with the lesion being located in the middle (14 eyes, 56%) and anterior (11 eyes, 44%) thirds of the corneal stroma. Corneal vascularization, blepharospasm, conjunctivitis, and excessive lacrimation were the associated clinical findings. All cases reported here had a history of short-term use of ophthalmic corticosteroids before presentation. The patients’ signalments and characterization of the sequestrum in each of the three groups are presented in Table 1.

### 3.2. Surgical Outcome

**Figure 6:**
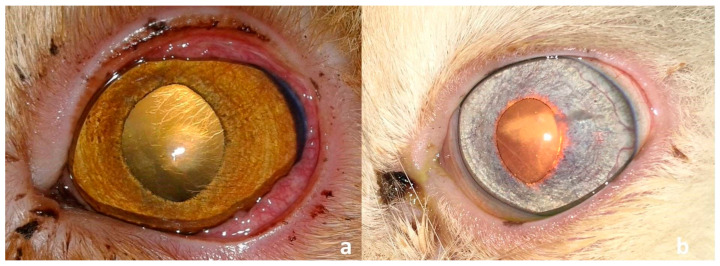
Clinical presentation of two cats treated with self-adhesive human bandage contact lens (SHBCL) showing complete healing of the corneal defect with light corneal scarring (**a**) and a moderate degree of corneal fibrosis (**b**).

The mean (±SD) recheck time after the initial presentation was 7.6 (±0.8) days, with a range of 6–9 days. In the G-SHBCL group, the contact lens retention rate was 80% (8/10 cats) at the first recheck examination. In the two cats with poor retention (20%), no corneal or conjunctival complications were observed following lens fallout; however, the loss compromised the intended protective function. To improve lens fixation, lateral tarsorrhaphy was performed and a new lens was applied in both cases. The second recheck examination meantime (±SD) was 15.7 (±1.2) days after the initial presentation, with a range of 14–18 days. At the second recheck examination, there were no complaints about lens retention or adhesion reported by the owners. The mean time (±SD) of the third recheck examination was 31.2 (±1.3) days after the initial presentation with a range of 29–34 days. By that time, 80% of the cats treated with SHBCL showed complete healing of the corneal defect with light corneal scarring and absence of all signs of ocular pain or discomfort (blepharospasm, redness, or discharge) (Figure 6a). However, the remaining 2 cats (20%) showed a moderate degree of corneal fibrosis and slight corneal vascularization (Figure 6b). Statistical analysis using Fisher’s Exact Test revealed no significant difference in the healing success rate between the SHBCL and CTF groups (*p* = 0.64). The detailed clinical outcomes of the three treatment groups at the three recheck visits are presented in Table 2. In the conjunctival transposition flap group (G-CTF), the conjunctival flap was removed at the third recheck.

**Figure 7:**
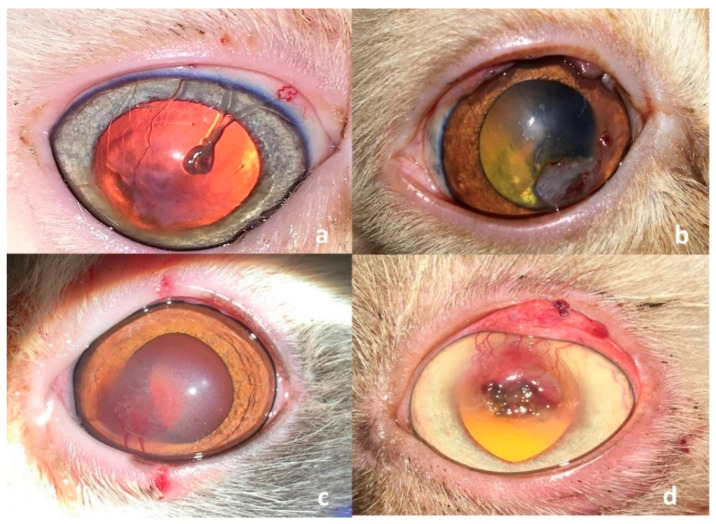
Clinical presentation of four cats treated with conjunctival transposition flap (CTF) showing corneal scarring (**a**,**b**), granulation tissue (**c**) and corneal granulation tissue with residues of sequestrum and fibrosis (**d**).

Three out of ten (30%) patients showed complete healing of the corneal defect with minimal to severe corneal scarring and vascularization (Figure 7a,b). A slight degree of granulation tissue formation and adhesion between the flapped conjunctival membrane and the cornea was observed in 50% of the cats (5/10 cats). The remaining two cats showed corneal granulation tissue with fibrosis and residues of the corneal sequestrum (Figure 7c,d).

**Figure 8:**
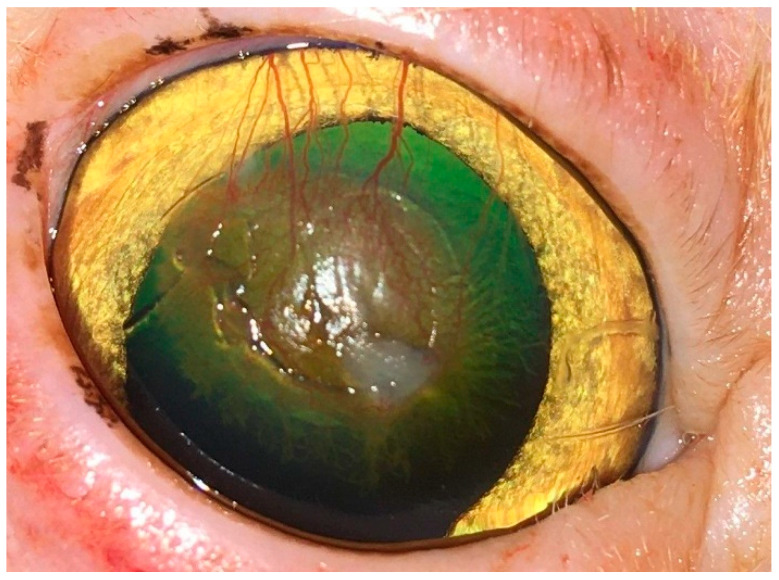
Clinical presentation of one cat from the control group showing incomplete healing of the corneal defect with corneal vascularization and scarring.

The corneal defects in the 5 cats included in the control group did not heal properly by the end of the fourth week postoperatively. These defects were manifested by the presence of moderate to severe degrees of corneal fibrosis and granulation tissue (Figure 8).

## 4. Discussion

Excision of corneal sequestrum usually leaves a corneal defect that requires a similar treatment procedure as other corneal ulceration. Over the past decades, various surgical techniques have been used for the management of deep-melting corneal ulcers or corneal sequestra. Despite these techniques being associated with a high success rate and a degree of restoration of corneal clarity, the quality of vision was variable [2]. Contact lens placement was described previously in veterinary medicine as an adjunct therapy for the treatment of superficial corneal ulcers in dogs and cats [18] and spontaneous chronic corneal epithelial defects in dogs [19]. Furthermore, contact lens placement was used to provide temporary comfort prior to entropion surgery [18]. To the best of the authors’ knowledge, contact lens placement does not appear to be previously utilized for the treatment of corneal sequestrum in cats. The present study is the first to utilize the commercially available human Polymacon contact lens for surgical repair of corneal sequestrum in cats to achieve corneal clarity and vision restoration and to overcome the complications associated with other techniques. The study included cats with large, firmly adhered corneal sequestra and excluded cats with small, elevated plaques in which the sequestrum can be simply grasped by a forceps and removed using topical analgesia, with the resulting corneal defect being left to heal by granulation tissue [20]. The commonly affected cat breeds reported in this study were Persian (n = 12, 48%), Himalayan (n = 8, 32%), and DSH (n = 5, 20%). This is relatively consistent with previous studies that reported a high incidence of corneal sequestrum in brachycephalic cats, with Persian having the highest reported incidence followed by Siamese, Burmese, Himalayan, and Domestic Shorthair breeds [20,21]. The average age at initial presentation was 37.6 months (range, 12–72 months) with no sex predisposition (13 males, 12 females). This is in agreement with previous studies which reported that feline corneal sequestrum can occur in all ages (excluding neonates) and both sexes [20,22]. Several procedures have been described to surgically repair corneal sequestrum in cats with a 100% success rate [2,4,14], with or without disease recurrence. These procedures included conjunctival flap transposition (CFT) (17 and 24 cats, up to 16.7% recurrence) [4,14], small intestinal submucosa (SIS) grafting (34 cats, 11.8% recurrence) [14], and keratectomy alone (44 cats, 2.3% recurrence) [2]. Amniotic membrane transplantation (AMT) accomplished a relatively high success rate and proper restoration of corneal transparency in 5 out of 7 (71.4%) treated eyes, with no recurrence of the disease after a 9-month follow-up period [23]. However, suppression of the myofibroblast differentiation and an increased level of matrix metalloproteinase activity can lead to corneal thinning and perforation after the use of AMT [11].

In a different study, Dulaurent et al. utilized a bovine pericardial (BP) graft for surgical repair of melting corneal ulcers in dogs and corneal sequestrum in cats with a high success rate [5]. However, the use of BP grafts remains limited for cases with infected corneal defects [5]. Moreover, the possibility of the pericardial graft being infected with the organism responsible for keratomalacia has been reported [5], with the possible development of secondary calcification as reported in human literature [24]. In addition to disease recurrence and graft rejection, other complications included residual discoloration following CFT (6%) and SIS grafting (60%), bacterial infection after AMT (14%), and corneal ectasia after BP grafting (33%) [2,4,5,12]. The high success rate and lack of disease recurrence after the self-adhesive human bandage contact lens (SHBCL) technique reported in the present clinical study is consistent with the outcomes accomplished after SIS grafting, AMT, and BP grafting. However, the SHBCL technique demonstrates superiority owing to its simplicity, absence of infection or corneal thinning, achievement of proper healing within a short follow-up period, and lack of major postoperative complications. In this study, success was assessed based on clinical indicators such as absence of blepharospasm, ocular redness, and discharge, along with complete epithelial healing confirmed by fluorescein staining. Although no formal pain scoring system or quantitative fluorescein scale was used, incorporation of such objective measures in future studies would further strengthen the evidence supporting SHBCL outcomes.

At the first and second recheck times following the conjunctival transposition flap (CTF) technique, ocular discharge (up to 90%), corneal vascularization (up to 70%), and corneal edema (up to 40%) were the most reported ophthalmic findings in the present study. This may be attributed to the intense inflammatory response to suture material along with the trauma created during the fixation of the conjunctival flap to the cornea. The incidence of ocular discharge and corneal edema was relatively reduced to 8/10 (80%) and 3/10 (30%) at the second recheck time, respectively. At the end of the follow-up period (3rd recheck after flap removal), a slight degree of granulation tissue with adhesion between the flapped conjunctival membrane and the corresponding cornea was observed in 50% of the cats. Furthermore, two cats (20%) showed corneal granulation tissue with fibrosis and residues of the corneal sequestrum at the surgery site. There was complete healing of the corneal defect but with minimal corneal scarring and vascularization observed in 30% of the cats enrolled in this group. In the CTF technique, the mechanism of corneal healing is promoted by the tectonic support and direct blood supply provided to the lesion by the flap that is filling the defect, together with the accompanied patient comfort [25]. Regarding the results of the self-adhesive human bandage contact lens group (G-SHBCL), the lens retention rate at initial presentation was 80%. The poor lens retention (premature lens loss) observed in the other two cats could be attributed to the large palpebral fissure together with the continuous movement of the corresponding eyelids. Our reported high retention rate is relatively consistent with a previous study that reported a retention rate of 86% during the treatment of corneal ulcers in 7 DSH cats [26]. The retention rate of the utilized self-adhesive human contact lens was superior to that of the veterinary lens [26,27]. The poor retention associated with veterinary bandage lenses may be attributed to their higher rigidity and greater thickness compared with self-adhesive human lenses. In addition, the possible entrapment of air bubbles beneath veterinary lenses represents another limitation to their use [17]. These characteristics can result in the lens becoming trapped between the eyelids, leading to premature loss [26]. In contrast, the softer, smaller, and thinner self-adhesive human lens used in this study allowed smooth movement of the third eyelid over the lens and demonstrated a good retention rate. However, despite these short-term advantages, potential long-term incompatibility of human lenses in cats should be considered, as differences in corneal curvature, tear film composition, and blink dynamics between humans and felines could predispose to lens instability, ocular surface irritation, or chronic inflammation with prolonged use. Future studies with extended follow-up periods are warranted to evaluate these potential effects. Ocular irritation was a common complication associated with wearing contact lenses in canine patients [26,27]. Contact lens intolerance was reported in five of 26 (19%) and one of 36 (3%) dogs wearing lenses for the treatment of chronic superficial keratitis and spontaneous chronic corneal epithelial defects, respectively [19,28]. The reported suspected causes were the entrapment of hairs between the lens and the cornea and suboptimal fit of the lens (contact lens-induced acute red eye) [17]. On the contrary, no ocular irritation was identified in all cats treated with SHBCT in the present study. Additionally, a successful outcome was obtained by the use of SHBCL as 80% of our enrolled cats showed complete healing of the corneal defect with light corneal scarring and absence of all signs of ocular pain or discomfort. The healing power accomplished by the self-adhesive contact lens was attributed to the ability of the lens to remove necrotic tissues and tissue residues by adhering them to the posterior lens surface. This mechanism would prevent the further destruction and melt of the cornea by tissue-destructing enzymes created by necrotic residues, thereby enhancing normal corneal repair [18].

Diamond burr debridement is a safe, non-invasive treatment for corneal ulceration in cats; however, it has a lower success rate than superficial lamellar keratectomy [29]. The bandage contact lens use and retention significantly improved the healing after diamond burr debridement (DBD) in dogs with spontaneous chronic corneal epithelial defects [30]. Unlike conjunctival transposition flap (CTF), the self-adhesive soft contact lens showed no granulation tissue or residues of corneal sequestrum or adhesion with a high degree of corneal clarity. In addition, all signs of ocular pain and discomfort associated with suturing of the conjunctival flap to the cornea are avoided. At the third recheck examination, all treatment groups showed negative fluorescein tests.

All cats in the present study had a history of prior topical corticosteroid use before referral, although corticosteroids were not part of our treatment protocol. The deleterious effects of corticosteroids on corneal healing are well documented, including delayed epithelialization, suppression of keratocyte activity, and potentiation of stromal collagenolysis [31]. These effects may have contributed to the progression of corneal sequestra observed at presentation. Awareness of corticosteroid-associated risks is therefore important in managing feline corneal disease, and their use should be avoided unless strongly indicated and closely monitored.

### Study Limitations

Corneoconjunctival transposition (CCT) was not utilized in the current study. This decision was based on the fact that the majority of the cases presented with extensive and deep stromal sequestra, often occupying a large portion of the corneal surface. In such advanced cases, CCT is generally considered suboptimal due to its limited ability to provide adequate tectonic support and coverage for large corneal defects. Instead, the utilized surgical techniques were prioritized to offer broader structural reinforcement and enhanced healing potential. The relatively short monitoring period may have precluded the detection of late-onset complications or long-term outcomes. In addition, the reduced sample size limits the statistical power and generalizability of the findings. Functional vision assessment was not performed, as tests such as the menace response, maze navigation, or obstacle avoidance were not included in the study design. Incorporating these evaluations in future studies would provide a more comprehensive assessment of visual recovery and functional outcomes following the procedure.

## 5. Conclusions

The presented SHBCL perfectly fits the cat’s eye and the corresponding corneal curvature. Application of SHBCL on corneal defects (after removal of corneal sequestrum) enhances healing of the cornea with efficient corneal transparency. The reported lens has been found to markedly reduce the risk of granulation tissue formation and fibrosis of the healed corneal defect that is potentially responsible for vision impairment.

## Figures and Tables

**Table 1:** Signalment of the enrolled three groups of cats and characterization of the sequestrum diagnosed in each group.

Group of Cats	No.	Breed	Patient Data Sex	Age (m)	Eye	Duration (m)	Site	Characterization of the Sequestrum Diameter (mm)	Depth
SHBCL (10 cats)	1	Persian	M	24	OD	2	CC	7	A 1/3
	2	Persian	M	28	OD	6	CC	4	M 1/3
	3	Himalayan	M	36	OS	7	CC	4	M 1/3
	4	Himalayan	F	34	OD	8	PC	5	M 1/3
	5	Himalayan	M	60	OD	12	CC	6	M 1/3
	6	Persian	F	42	OS	12	CC	8	A 1/3
	7	Himalayan	M	54	OS	5	CC	6	M 1/3
	8	Persian	F	16	OS	7	PC	5	M 1/3
	9	Persian	M	24	OS	8	PC	6	A 1/3
	10	DSH	F	42	OD	8	CC	4	A 1/3
CTF (10 cats)	1	Himalayan	M	36	OS	9	CC	6	M 1/3
	2	DSH	F	54	OD	12	CC	5	M 1/3
	3	Himalayan	F	72	OS	14	CC	6	A 1/3
	4	Persian	M	36	OD	14	CC	4	M 1/3
	5	Persian	F	12	OS	12	CC	5	M 1/3
	6	Himalayan	M	28	OS	14	CC	7	M 1/3
	7	DSH	M	46	OD	10	CC	5	M 1/3
	8	Persian	F	24	OD	12	PC	4	A 1/3
	9	DSH	M	24	OD	8	PC	7	A 1/3
	10	Persian	F	54	OD	2	PC	4	A 1/3
CO (5 cats)	1	DSH	M	72	OS	4	CC	4	M 1/3
	2	Persian	M	46	OS	12	CC	6	M 1/3
	3	Himalayan	F	18	OD	6	PC	5	A 1/3
	4	Persian	F	24	OD	12	PC	7	A 1/3
	5	Persian	F	36	OD	14	CC	8	A 1/3

SHBCL, self-adhesive human bandage contact lens; CTF, conjunctival transposition flap; CO, control; M, Male; F, Female; DSH, Domestic shorthaired; OD, Oculus dexter (right eye); OS, Oculus sinister (left eye); CC, central cornea; PC, Paracentral; A 1/3, Anterior third of the stroma; M 1/3, Middle third of the stroma.

**Table 2:** Ophthalmic findings at the first, second, and third recheck examination for the three treatment groups (25 cats).

Ophthalmic Findings	First Recheck Exam (7.6 ± 0.8 Days)			Second Recheck Exam (15.7 ± 1.2 Days)			Third Recheck Exam (31.2 ± 1.3 Days)		
	G-SHBCL (n = 10)	G-CTF (n = 10)	G-CO (n = 5)	G-SHBCL (n = 10)	G-CTF (n = 10)	G-CO (n = 5)	G-SHBCL (n = 10)	G-CTF (n = 10)	G-CO (n = 5)
Ocular discharge	6 (60%)	9/10 (90%)	5 (100%)	None	8 (80%)	5 (100%)	None	7 (70%)	5 (100%)
Blepharospasm	3 (30%)	4/10 (40%)	4 (80%)	None	2 (20%)	4 (80%)	None	None	4 (80%)
Corneal vascularization	2 (20%)	9/10 (45%)	5 (100%)	None	7 (70%)	4 (80%)	2 (20%)	2 (20%)	4 (80%)
Corneal edema	3 (30%)	8/10 (40%)	3 (60%)	2 (20%)	3 (30%)	4 (80%)	None	None	2 (40%)
Squinting	1 (10%)	4/10 (40%)	None	5 (50%)	None	None	None	None	None
Residues of the sequestrum	4 (40%)	NA	2 (40%)	2 (20%)	NA	2 (40%)	None	2 (20%)	2 (40%)
Granulation tissue	None	NA	4 (80%)	None	NA	5 (100%)	None	5 (50%)	5 (100%)
Corneal scarring	None	NA	3 (60%)	3 (30%)	NA	5 (100%)	2 (20%)	2 (20%)	5 (100%)
Positive fluorescein test	None	NA	2 (40%)	None	NA	None	None	None	None

G, group; SHBCL, self-adhesive human bandage contact lens; CTF, conjunctival transposition flap; CO, control; NA, not applicable (due to the covering flap).

## Data Availability

The data is available from the corresponding authors upon request.

## Abbreviations

SHBCL	self-adhesive, human bandage contact lens
CTF	conjunctival transposition flap
G-CO	control group
FHV-1	feline herpesvirus type-1
DSH	domestic short hair
SIS	small intestinal submucosal
AMT	amniotic membrane transplantation
BP	bovine pericardial
